# A Matter of Botanic and Zoologic Analysis

**Published:** 1916-06

**Authors:** 


### BUFFALO MEDICAL JOURNAL

**A Monthly Review of Medicine and Surgery**

EDITOR AND PUBLISHER, DR. A. L. BENEDICT, 228 Summer St., corner of Elmwood Ave., Buffalo, (Address for all communications. Please make personal and telephone calls before 1 P. M.)

**Third, in age, on the western continent. Independent, but supports professional organization. News limited mainly to Western N. Y. and Penn.**

**Subscription $2.00 a year; $3.00 for two years in advance. Changes and errors in address or failure or duplication of delivery, should be reported immediately.**

Yearly Volume 71 JUNE, 1916 No. 11

### A Matter of Botanic and Zoologic Analysis.

A large and important group of diseases are caused by germs. We use this term because, when one checks off the list, he is surprised to find how many pathogenic organisms are animal and how closely analogous are the infections caused by vegetable and animal organisms. The term is objectionable because used in different senses and the term micro-organism is objectionable because there is no sharp dividing line between very minute and fairly large organisms, indeed, there is no conspicuous division as one progresses from infections supposed to be due to ultramicroscopic organisms up to a 20-foot tape worm through such conditions as trichinosis, however marked the contrast between the typic infection and the ordinary infestment with a "parasite" in the limited sense.

We have searched in vain for general characteristics that would separate diseases due to bacteria from those due to other vegetable germs such as the actinomyces, and the rather vaguely described organisms of sporotrichosis, etc., those due to minute protozoa, such as malaria, and those due to macroscopic but minute animal parasites or larval forms of larger parasites. In the case of many minute germs, more or less definitely associated with specific infections, the assumption of vegetable or animal nature seems to be without actual demonstration, if indeed the two kingdoms do not actually merge in undifferentiated organisms and, in regard to spiral forms, there seems to be either a lack of positive discrimination between animal and vegetable types or, at least, a marked analogy between infections caused by the two types.

Our attention has been called to the urgent need of exact methods of determination or organisms, along lines of botanic or zoologic classification, and for purely practical purposes, by an article by Dr. Arthur I. Kendall of Chicago in the Boston M. & S. Jour. of Nov. 20, 1913. He reports two cases of mild diarrhoea and fever in twins, one presenting Shiga dysentery bacilli, with beta paratyphoid, yeast and streptococci, the other Flexner bacilli, yeast, streptococci and the Morgan bacillus. He also reports a similar mild case in a child who yielded Shiga and Morgan bacilli.

Not to dwell on the occurrence of dysentery bacilli without typic dysentery, the concurrence of an infection in children so closely associated as twins, with practically identical disease but differing types of intestinal flora, is remarkable—and we use the word **remarkable** without implying exceptional.

Essentially the same problem of germ identification occurs in many widely different cases, and as the author implies, whether reliance is placed on cultural and tinctorial features or biologic reactions. Even so familiar and easily demonstrated a germ as the bacillus tuberculosis is either liable to confusion with innocuous germs, as in smegma and faeces, or it exists in spontaneously acquired habitats in extreme degrees of difference of infectiousness, not to mention that it has been reduced to practically innocuous types by long cultivation.

Excluding gross errors in diagnosis, atypic symptomatology, etc., it must still be admitted that a clinical infection cannot invariably be connected with a definite germ. For example, it is only apparently incorrect to speak of "pneumonia of the lungs" if, by pneumonia, we mean a pneumococcic infection nor, if by the word we refer to morbid anatomy, to speak of pneumococcic pneumonia. The word diphtheria, as used twenty years ago not only excluded mild infections with the diphtheria bacillus but included processes due to other bacteria—a very practical point to remember in estimating the value of antitoxin.

Kendall's article suggests an interesting question as to clinical definition. It is universally conceded that contact with specific germs does not necessarily produce a disease reaction and that the extent of the reaction does not correspond mathematically either with the extent of the lesion nor with the number of germs introduced or developed. Yet professional opinion is singularly inconsistent in the practical application of these principles. For example, it cannot be questioned that diphtheria may vary from a colonization of bacilli with no appreciable reaction, through mild sore throats, up to the most malignant types. Considerable variations in intensity of reaction are recognized in typhoid but one would have to be very bold to report a case of a week's duration, or to use the word abortive, much less aborted. On the other hand, clinical typhoid is certainly occasionally and, possibly quite frequently, due to para-bacilli and the specific bacillus probably evolved from the colon bacillus, through para-forms, at a date subsequent to the separation of the American race from the parent stock, if it did not develop primarily in some European race.

But, without reference to the evolution of various human infections subsequent to the development of man, to the much later evolution of various local infections at a much later date, and without regard to the fact that a clinical symptom complex may be due to various germs or, conversely, that the same infectious process may occur in varying type, the general principle that a well defined disease is due to a definite germ, probably holds good.

It would, therefore, be a decided advantage if the positive identification of disease germs could be made, independently of clinical manifestations, along lines analogous to those followed in the identification of macroscopic plants and animals. It is unnecessary to apologize for the failure of any branch of medical science to have reached a state of relative perfection. All things considered, we may rather congratulate ourselves that we have advanced so far as we have and this is particularly true of bacteriology (using the term in an inclusive sense), considering its newness. But it may not be out of place to point out that the systematic analysis of a minute, unicellular organism, presents difficulties far greater than analysis applied to complex plants and animals. Last summer, we found and used a bush blue berry that did not correspond exactly to any of the species described by Wood and Gray, and it is not surprising that the bacteria and minute fungi, protozoa, etc., whose economic interest is limited to the harm that they may do, are still described in a fragmentary and rudimentary way.

